# Beyond the diagnostic threshold: paradigm shift in laboratory medicine from passive reaction to predictive capability

**DOI:** 10.3389/fmed.2026.1781957

**Published:** 2026-05-21

**Authors:** Yu Zhang, Li Dong, Yun-xia Xiu, Cong Shen

**Affiliations:** 1 Clinical Laboratory, The Second Affiliated Hospital of Mudanjiang Medical University, Mudanjiang, China; 2 Department of General Surgery, Hongqi Hospital Affiliated to Mudanjiang Medical University, Mudanjiang, China

**Keywords:** artificial intelligence, diagnostic thresholds, laboratory medicine, multi-omics integration, precision medicine, predictive paradigm

## Abstract

Conventional laboratory medicine, which relies on diagnostic thresholds, operates within a reactive framework that is inherently constrained by its retrospective and static nature. This perspective article scrutinizes the paradigm shift in the field from a responsive to a predictive discipline. The transition is centered on integrating multi-omics data, continuous biosensing, and artificial intelligence to build predictive systems capable of early risk identification and individualized health guidance. The perspective article further investigates the conceptual underpinnings, implementation pathways, and related challenges—spanning technical, ethical, and health-system dimensions—of this evolving paradigm. This transformation not only redefines the scope of laboratory medicine but also positions it as a foundational component within precision medicine and prevention-focused public health frameworks.

## 1 Introduction

Laboratory medicine has traditionally operated on a “post-facto verification” model. Its primary function has been to confirm or rule out clinical suspicions by comparing measured values against fixed diagnostic thresholds (1). Although effective for diagnosing and staging overt disease, this reactive paradigm has inherent limitations (2). It provides a static, cross-sectional snapshot rather than a dynamic assessment of individual risk (3). Furthermore, its reliance on population-based thresholds often fails to capture the biological continuum between health and disease, and its reports tend to offer isolated data points rather than integrated pathophysiological insight (4, 5).

A convergence of forces is now driving a fundamental transformation of this role. The rise of precision medicine demands strategies tailored to individual risk profiles, not population averages (6). Advances in multi-omics technologies—genomics, proteomics, metabolomics—enable systematic interrogation of disease mechanisms at a molecular level (7, 8). Meanwhile, the integration of big-data analytics and artificial intelligence allows the extraction of predictive signals from complex, high-dimensional datasets (7, 9). Underlying these technological shifts is a broader reorientation in healthcare: from reactive disease treatment toward proactive health maintenance and early intervention (7, 10).

**Figure 1:**
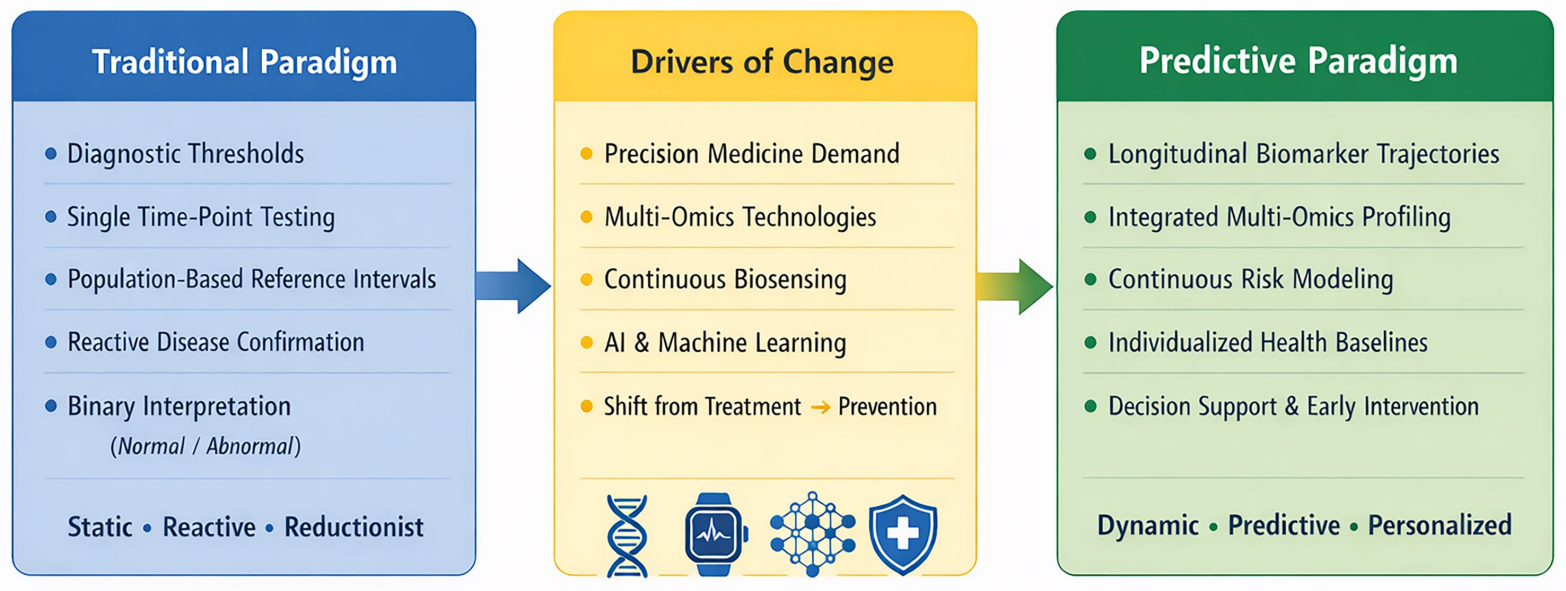
Paradigm shift in laboratory medicine: from threshold-based diagnostics to predictive health management.

In light of this transition, this perspective article elaborates the shift of laboratory medicine from a passive-responsive service to a predictive discipline (Figure 1). We first scrutinize the epistemological constraints of the traditional threshold-based model. Next, we outline the logical imperatives for change and describe the key components of a predictive paradigm—including continuous biomarker monitoring, integrated multi-omics profiling, and algorithmic risk modeling—along with their implications for reporting and clinical decision-support. Finally, we discuss critical challenges such as data standardization, ethical governance, clinical validation, and workforce development that should be addressed to realize a future in which laboratory medicine serves as a cornerstone of predictive health management.

## 2 Traditional paradigm: passive-responsive model centered on diagnostic thresholds

### 2.1 Philosophical basis and operational logic

The traditional paradigm of laboratory medicine is a passive-responsive model fundamentally organized around diagnostic thresholds. For decades, this framework has served as the foundation of clinical diagnostics. Its approach is philosophically grounded in a dichotomous logic derived from population statistics, wherein reference intervals are established from large cohorts of presumed healthy individuals (11). Diagnostic cut-off values are subsequently treated as a “gold standard” to categorically separate “normal” from “abnormal” results, thereby distinguishing “health” from “disease” (12). Operationally, this model follows a straightforward rule: a patient’s result is compared to these predetermined thresholds to support or reject a clinical hypothesis (2, 13).

### 2.2 Strengths and contributions

This paradigm offers significant advantages, primarily through standardization and operational efficiency (14). Uniform procedures, consistent reference intervals, and clear decision rules ensure reproducible results across different laboratories and over time, providing clinicians with reliable, actionable information (15). It has proven essential for diagnosing and classifying established diseases, assessing their severity, and objectively monitoring treatment response (11, 16). Its structural simplicity allows for effective implementation even in resource-constrained settings (17).

### 2.3 Inherent limitations

Despite its utility, the model possesses critical inherent limitations. First, it is inherently reactive; testing is usually initiated only after symptoms appear, confirming established biological changes rather than predicting risk (18). Second, it is static, relying on single-point measurements that fail to reveal an individual’s biomarker trajectory over time (19). Third, it carries a risk of population bias, as thresholds derived from group averages may not accurately reflect the biology of unique individuals—particularly those whose values lie near the reference limits (20). Most fundamentally, it oversimplifies complex pathophysiology by reducing multifaceted disease processes to binary outcomes based on isolated metrics (21). This reductionist approach neglects the integrated and adaptive nature of human physiology. Collectively, these shortcomings highlight the paradigm’s growing misalignment with the forward-looking, individualized demands of precision health and proactive care.

## 3 Predictive paradigm: conceptual core and theoretical framework

### 3.1 Definition and objectives

The predictive paradigm marks a fundamental transition for laboratory medicine, shifting its focus from diagnosing established disease to prospectively evaluating health trajectories (22) (Table 1). Its primary objective is to generate comprehensive assessments of an individual’s future disease risk, probable progression pathways, and potential therapeutic responses (23). This is achieved by synthesizing longitudinal data, multidimensional biomarker profiles, and advanced computational models (24). Moving beyond static diagnostic categorization, the paradigm seeks to detect risk before clinical manifestation, thereby enabling truly preventative and personalized care (25).

**Table 1:** Comparison between traditional diagnostic and predictive paradigms in laboratory medicine.

Dimension	Traditional paradigm	Predictive paradigm	Key technologies	Clinical implication
Data type	Single biomarkers	Multi-omics plus longitudinal	Omics, wearables	Early risk detection
Time scale	Cross-sectional	Continuous/dynamic	Sensors, EHR	Trajectory monitoring
Interpretation	Threshold-based	Probability-based	AI/ML models	Risk stratification
Reference	Population intervals	Personal baseline	Digital twins	Individualized care
Clinical role	Diagnostic support	Decision guidance	CDS systems	Prevention-oriented care

AI, artificial intelligence; ML, machine learning; EHR, electronic health records; CDS, clinical decision support.

For instance, in the context of cardiometabolic diseases, predictive models integrate longitudinal data on lipid profiles, glycaemic markers, inflammatory indices, and polygenic risk scores to stratify individuals according to future risk, often well before conventional diagnostic thresholds are met (26, 27). This pre-clinical risk stratification facilitates the timely initiation of tailored lifestyle modifications or pharmacological interventions (28, 29). Consequently, predictive laboratory analytics directly inform and guide preventive clinical decision-making, transcending the traditional role of merely confirming established pathology (27).

### 3.2 Theoretical pillars

The theoretical basis of this approach is supported by three interrelated pillars. The first is a systems biology perspective, which frames laboratory markers as components within broader biological networks—such as signaling, metabolic, and regulatory pathways—rather than as isolated measures (30, 31). This network-aware viewpoint allows the detection of subtle, early systemic disturbances that precede overt pathology. The second pillar emphasizes individualized dynamic monitoring, which involves establishing a personalized, time-series health baseline and tracking deviations from it over time (32). Such deviations often signal the earliest phases of subclinical disease. The third pillar replaces binary classification with a continuous risk model, in which health status is represented along a probability gradient rather than as “normal” or “abnormal” (33, 34). This permits refined risk stratification and supports the definition of context-sensitive intervention thresholds.

### 3.3 Key technological enablers

Operationalizing this paradigm relies on converging technological advances. Integrated multi-omics profiling-spanning genomics, proteomics, metabolomics, and microbiomics-provides a high-resolution, multidimensional portrait of an individual’s physiological state (6, 35). Continuous sensing and wearable technologies deliver real-time, streaming physiological data, enabling the construction of dynamic health trajectories (36). Finally, artificial intelligence and machine learning techniques uncover complex patterns within these high-dimensional datasets, facilitate predictive modeling, and support individualized risk forecasting (6, 36). Together, these technologies form the infrastructure necessary to translate predictive concepts into clinical practice. Nevertheless, significant challenges remain, including data standardization, model validation, ethical oversight, and the integration of predictive insights into existing clinical workflows, all of which require deliberate and coordinated resolution (37, 38).

## 4 Implementation pathways for paradigm restructuring

The successful transition from a reactive to a predictive paradigm in laboratory medicine necessitates coordinated transformation across three interdependent domains: data, analytics, and clinical practice. This restructuring represents a fundamental evolution in the discipline’s operational workflow, professional identity, and contribution to patient care.

### 4.1 Data-level transformation

At the data level, the core objective is to expand from static, isolated measurements to integrated, longitudinal datasets (39). This involves moving beyond traditional single- or few-biomarker snapshots toward the systematic aggregation of multidimensional and multi-omics information—encompassing genomic, proteomic, and metabolomic data, contextualized with electronic health records and behavioral inputs (39, 40). Crucially, data should evolve in form from discrete reports into continuous streams, enabled by wearable sensors and persistent monitoring technologies (41, 42). This shift facilitates the creation of individualized longitudinal health trajectories, providing the temporal resolution needed to identify subtle, preclinical biological deviations (42, 43).

### 4.2 Analysis-level transformation

At the analytical level, the focus shifts from passive interpretation to active, model-enabled insight generation. Analytical processes should advance from binary threshold comparisons to algorithmically driven predictive modeling using machine learning and artificial intelligence (44). Such models can synthesize diverse data to produce individualized outputs—for example, quantitative risk scores, prognostic estimates, or treatment response predictions (44). Furthermore, the analytical product should transcend simple result reporting to offer integrative interpretation and clinical decision support (45). This entails situating data within a patient-specific clinical narrative, explaining its biological significance, and suggesting actionable steps based on stratified risk.

### 4.3 Clinical practice-level transformation

At the clinical practice level, transformation redefines roles, outputs, and collaborations. Laboratory professionals will expand their scope from technical oversight to roles as integrative data consultants and predictive analysts (46). They will be responsible for interpreting complex biomarker profiles, translating model outputs into actionable clinical context, and advising on result implications for individual patient management (47). This evolution in roles also calls for a redefinition of professional competencies (48). In addition to technical proficiency in assays, laboratory professionals will need to strengthen their skills in data interpretation, risk communication, interdisciplinary collaboration, and the critical evaluation of predictive models (49). Accordingly, training programs should incorporate data literacy, clinical consultation skills, and ethical awareness (50). This shift highlights that the move toward predictive diagnostics is not merely technological, but also involves organizational and educational transformation.

Concurrently, this transformation demands innovations in reporting formats. Traditional text-based summaries will be augmented by interactive, visual tools such as dynamic trend visualizations, risk-probability diagrams, and context-aware clinical guidance (51, 52). Ultimately, the paradigm’s success hinges on deepened interdisciplinary collaboration (51). Laboratory medicine should actively partner with clinical specialties, bioinformatics, data science, and public health to co-develop, validate, and implement predictive tools, ensuring their effective integration into holistic care pathways for prevention, diagnosis, and management (51).

## 5 Challenges and countermeasures

The implementation of a predictive paradigm in laboratory medicine encounters significant challenges across technical, ethical-regulatory, and healthcare-system domains. A systematic and coordinated response is essential to enable its responsible integration into clinical practice.

### 5.1 Technical challenges

Key technical obstacles include data standardization, model interpretability, generalization, and biomarker validation. Effective data integration requires uniform protocols for collection, formatting, and exchange (53). Many advanced predictive models, particularly deep learning systems, function as “black boxes,” limiting clinical trust due to poor transparency (54). Furthermore, models trained on narrow datasets often fail to generalize across diverse populations or settings (55, 56). Finally, the discovery and validation of novel biomarkers remain protracted and costly, requiring rigorous biological and clinical substantiation (57).

### 5.2 Ethical and regulatory challenges

Predictive analytics introduces ethical complexity, particularly regarding uncertainty, privacy, and liability. Communicating probabilistic risk—especially intermediate probabilities—may cause patient anxiety or prompt unnecessary interventions, underscoring the need for careful risk communication frameworks (58). Large-scale, longitudinal health data collection raises substantial privacy and security concerns, demanding robust governance (59). Ambiguity also exists around accountability for decisions based on predictive outputs, such as insurance or employment rulings, highlighting the necessity of clearer legal and regulatory guidance (60).

### 5.3 Healthcare system integration challenges

Integrating predictive tools requires adaptation across reimbursement, clinical workflow, and workforce readiness. Current payment models rarely compensate preventive risk assessment, creating a financial disincentive for adoption (61). Clinicians may lack familiarity with interpreting predictive reports, necessitating training and decision-support integration (62, 63). Laboratory professionals, in turn, should expand their competencies beyond analytical testing to include data interpretation, model evaluation, and clinical consultation (64).

Importantly, the feasibility and speed of this integration will likely differ substantially across healthcare systems with heterogeneous resources (65). Settings with limited resources may encounter amplified challenges related to foundational digital infrastructure, workforce capacity for new skill acquisition, and the development of viable reimbursement mechanisms (66, 67). This variability underscores the critical importance of developing tailored, context-sensitive implementation strategies, rather than adopting a uniform, one-size-fits-all global approach (68).

### 5.4 Comprehensive countermeasures

A multifaceted strategy is required: technically, implementing phased real-world validation and developing industry standards for data and algorithms (69, 70); ethically, promoting transparent communication and shared decision-making (71); and systemically, aligning payment models with preventive care and supporting workforce development through education and role redesign (72). Policy initiatives and interdisciplinary collaboration will be critical to ensuring that the predictive paradigm is translated into safe, equitable, and clinically meaningful practice (73).

## 6 Future perspectives

The continued evolution of laboratory medicine toward a predictive paradigm is expected to proceed through distinct, progressive stages, advancing from targeted clinical applications to a foundational component of population health management.

### 6.1 Near-term outlook

In the near term, efforts will concentrate on translating validated predictive models into clinical use for specific high-prevalence conditions (74). This will involve implementing risk-stratification tools for diseases with well-characterized risk continua, such as diabetes, cardiovascular disorders, and certain cancers (75). During this phase, laboratory medicine can first demonstrate its value in conditions where longitudinal biomarker monitoring is already part of routine care. This provides a practical entry point for the broader adoption of predictive approaches. These models will synthesize conventional biomarkers, genetic data, and emerging multi-omics markers to guide early risk detection and individualized screening, supported by evolving clinical guidelines (74, 76). Success during this phase will depend on completing prospective clinical trials, demonstrating utility in real-world settings, and establishing clear protocols for interpretation and clinical response (74, 77).

### 6.2 Medium-term horizon

In the medium term, emphasis will shift toward developing integrated personal health data ecosystems. These platforms will unify longitudinal information from multiple sources—including laboratory results, data from wearable sensors, genomic profiles, and environmental exposures—to generate dynamic, individualized health portraits (78). Analytics will expand beyond single-disease prediction to enable continuous, lifespan health risk monitoring (79). This approach will support earlier detection of multifactorial chronic conditions and provide an evidence base for personalized health maintenance and preventive interventions (80).

### 6.3 Long-term vision

In the long term, laboratory medicine is positioned to evolve from a diagnostic support service into a central element of a proactive health intelligence system (81). Its role will extend across the continuum of care—from health promotion and primary prevention to chronic disease management. By providing continuous, interpretable, and actionable health risk insights, the discipline will facilitate closer collaboration across clinical, public health, and community-based care (82). This transition will help shift healthcare systems from a reactive, treatment-focused model to a sustainable, prevention-oriented framework, ultimately contributing to the goals of precision public health and equitable health outcomes (83, 84).

## 7 Summary

The paradigm shift in laboratory medicine constitutes a fundamental transition from its role as a diagnostic tool to its emergence as a predictive engine. This evolution extends, rather than replaces, the value of traditional diagnostics by integrating advanced methodologies—such as multi-omics, continuous monitoring, and artificial intelligence—into a more comprehensive framework for understanding health and disease.

Realizing this shift demands coordinated advancement in four key areas: technological innovation, clinical integration, ethical governance, and systemic support. Interdisciplinary collaboration is essential to develop rigorously validated predictive models, implement them within redesigned clinical workflows, and establish aligned regulatory and reimbursement frameworks.

In the long term, predictive laboratory medicine will facilitate a broader transition in healthcare—from reactive treatment toward proactive health management. By generating individualized, forward-looking insights, it will empower earlier interventions and reshape the healthcare value chain across prevention, risk stratification, and precision care. Ultimately, this evolution will help build a more sustainable, prevention-oriented health system.

## Data Availability

The original contributions presented in the study are included in the article/supplementary material, further inquiries can be directed to the corresponding author.

## References

[1] Coskun A Sandberg S Unsal I Serteser M Aarsand AK. Personalized reference intervals: from theory to practice. Crit Rev Clin Lab Sci. (2022) 59:501–16. doi: 10.1080/10408363.2022.2070905, 35579539

[2] Timbrell NE. The role and limitations of the reference interval within clinical chemistry and its reliability for disease detection. Br J Biomed Sci. (2024) 81:12339. doi: 10.3389/bjbs.2024.12339, 38481978 PMC10932992

[3] Ozarda Y Sikaris K Streichert T Macri J. IFCC committee on reference intervals and decision limits (C-RIDL). Distinguishing reference intervals and clinical decision limits - a review by the IFCC committee on reference intervals and decision limits. Crit Rev Clin Lab Sci. (2018) 55:420–31. doi: 10.1080/10408363.2018.1482256, 30047297

[4] Buonpane A Trimarchi G Di Muro FM Nardi G Ciardetti M Coceani MA . From vision to illumination: the promethean journey of optical coherence tomography in cardiology. J Clin Med. (2025) 14:5451. doi: 10.3390/jcm14155451, 40807072 PMC12346916

[5] Parker LA Chilet-Rosell E Hernández-Aguado I Pastor-Valero M Gea S Lumbreras B. Diagnostic biomarkers: are we moving from discovery to clinical application? Clin Chem. (2018) 64:1657–67. doi: 10.1373/clinchem.2018.292854 30213783

[6] Srivastava R. Applications of artificial intelligence multiomics in precision oncology. J Cancer Res Clin Oncol. (2023) 149:503–10. doi: 10.1007/s00432-022-04161-4, 35796775 PMC11797275

[7] Ooka T. The era of preemptive medicine: developing medical digital twins through omics, IoT, and AI integration. JMA J. (2025) 8:1–10. doi: 10.31662/jmaj.2024-0213, 39926086 PMC11799569

[8] Chang L Liu J Zhu J Guo S Wang Y Zhou Z . Advancing precision medicine: the transformative role of artificial intelligence in immunogenomics, radiomics, and pathomics for biomarker discovery and immunotherapy optimization. Cancer Biol Med. (2025) 22:1–15. doi: 10.20892/j.issn.2095-3941.2024.0376, 39749734 PMC11795263

[9] He X Liu X Zuo F Shi H Jing J. Artificial intelligence-based multi-omics analysis fuels cancer precision medicine. Semin Cancer Biol. (2023) 88:187–200. doi: 10.1016/j.semcancer.2022.12.009, 36596352

[10] Abdelhalim H Berber A Lodi M Jain R Nair A Pappu A . Artificial intelligence, healthcare, clinical genomics, and pharmacogenomics approaches in precision medicine. Front Genet. (2022) 13:929736. doi: 10.3389/fgene.2022.929736, 35873469 PMC9299079

[11] Boyd JC. Defining laboratory reference values and decision limits: populations, intervals, and interpretations. Asian J Androl. (2010) 12:83–90. doi: 10.1038/aja.2009.9, 20111086 PMC3739683

[12] Jones HE Gatsonsis CA Trikalinos TA Welton NJ Ades AE. Quantifying how diagnostic test accuracy depends on threshold in a meta-analysis. Stat Med. (2019) 38:4789–803. doi: 10.1002/sim.8301, 31571244 PMC6856843

[13] Whyte MB Kelly P. The normal range: it is not normal and it is not a range. Postgrad Med J. (2018) 94:613–6. doi: 10.1136/postgradmedj-2018-135983, 30425140 PMC6352401

[14] Miller WG Tate JR Barth JH Jones GR. Harmonization: the sample, the measurement, and the report. Ann Lab Med. (2014) 34:187–97. doi: 10.3343/alm.2014.34.3.187, 24790905 PMC3999316

[15] Ozarda Y. Reference intervals: current status, recent developments and future considerations. Biochem Med (Zagreb). (2016) 26:5–16. doi: 10.11613/BM.2016.001, 26981015 PMC4783089

[16] Plebani M Sciacovelli L. ISO 15189 accreditation: navigation between quality management and patient safety. J Med Biochem. (2017) 36:225–30. doi: 10.1515/jomb-2017-0038, 30564060 PMC6287216

[17] Moussy FG Berumen AV Pai M. The WHO list of essential *in vitro* diagnostics: development and next steps. EBioMedicine. (2018) 37:1–2. doi: 10.1016/j.ebiom.2018.10.070, 30389503 PMC6286302

[18] Mayeux R. Biomarkers: potential uses and limitations. NeuroRx. (2004) 1:182–8. doi: 10.1602/neurorx.1.2.182, 15717018 PMC534923

[19] White E. Measurement error in biomarkers: sources, assessment, and impact on studies. IARC Sci Publ. (2011) 163:143–61. 22997860

[20] Petersen PH Jørgensen LG Brandslund I De Fine ON Stahl M. Consequences of bias and imprecision in measurements of glucose and hba1c for the diagnosis and prognosis of diabetes mellitus. Scand J Clin Lab Invest Suppl. (2005) 240:51–60. doi: 10.1080/00365510500236135, 16112960

[21] Ahn AC Tewari M Poon CS Phillips RS. The limits of reductionism in medicine: could systems biology offer an alternative? PLoS Med. (2006) 3:e208. doi: 10.1371/journal.pmed.0030208, 16681415 PMC1459480

[22] Collins FS Varmus H. A new initiative on precision medicine. N Engl J Med. (2015) 372:793–5. doi: 10.1056/NEJMp1500523, 25635347 PMC5101938

[23] Hood L Friend SH. Predictive, personalized, preventive, participatory (P4) cancer medicine. Nat Rev Clin Oncol. (2011) 8:184–7. doi: 10.1038/nrclinonc.2010.227, 21364692

[24] Chen R Mias GI Li-Pook-Than J Jiang L Lam HY Chen R . Personal omics profiling reveals dynamic molecular and medical phenotypes. Cell. (2012) 148:1293–307. doi: 10.1016/j.cell.2012.02.009, 22424236 PMC3341616

[25] Auffray C Charron D Hood L. Predictive, preventive, personalized and participatory medicine: back to the future. Genome Med. (2010) 2:57. doi: 10.1186/gm178, 20804580 PMC2945014

[26] Khera AV Chaffin M Aragam KG Haas ME Roselli C Choi SH . Genome-wide polygenic scores for common diseases identify individuals with risk equivalent to monogenic mutations. Nat Genet. (2018) 50:1219–24. doi: 10.1038/s41588-018-0183-z, 30104762 PMC6128408

[27] Arnett DK Blumenthal RS Albert MA Buroker AB Goldberger ZD Hahn EJ . 2019 ACC/AHA guideline on the primary prevention of cardiovascular disease: a report of the American College of Cardiology/American Heart Association task force on clinical practice guidelines. Circulation. (2019) 140:e596–646. doi: 10.1161/CIR.0000000000000678, 30879355 PMC7734661

[28] Khera AV Emdin CA Drake I Natarajan P Bick AG Cook NR . Genetic risk, adherence to a healthy lifestyle, and coronary disease. N Engl J Med. (2016) 375:2349–58. doi: 10.1056/NEJMoa1605086, 27959714 PMC5338864

[29] Tuomilehto J Lindström J Eriksson JG Valle TT Hämäläinen H Ilanne-Parikka P . Prevention of type 2 diabetes mellitus by changes in lifestyle among subjects with impaired glucose tolerance. N Engl J Med. (2001) 344:1343–50. doi: 10.1056/NEJM200105033441801 11333990

[30] Sonachalam M Shen J Huang H Wu X. Systems biology approach to identify gene network signatures for colorectal cancer. Front Genet. (2012) 3:80. doi: 10.3389/fgene.2012.00080 22629282 PMC3354560

[31] Hasin Y Seldin M Lusis A. Multi-omics approaches to disease. Genome Biol. (2017) 18:83. doi: 10.1186/s13059-017-1215-1, 28476144 PMC5418815

[32] Kotorov R Chi L Shen M. Personalized monitoring model for electrocardiogram signals: diagnostic accuracy study. JMIR Biomed Eng. (2020) 5:e24388. doi: 10.2196/24388, 33529270 PMC7814508

[33] Tomašev N Harris N Baur S Mottram A Glorot X Rae JW . Use of deep learning to develop continuous-risk models for adverse event prediction from electronic health records. Nat Protoc. (2021) 16:2765–87. doi: 10.1038/s41596-021-00513-5, 33953393

[34] Royston P Sauerbrei W Altman DG. Modeling the effects of continuous risk factors. J Clin Epidemiol. (2000) 53:219–20. doi: 10.1016/s0895-4356(99)00163-8, 10755886

[35] Theodorakis N Feretzakis G Tzelves L Paxinou E Hitas C Vamvakou G . Integrating machine learning with multi-omics Technologies in Geroscience: towards personalized medicine. J Pers Med. (2024) 14:931. doi: 10.3390/jpm14090931, 39338186 PMC11433587

[36] Jeong I Kong S Kim Y Kim Y Kim B Ahn SJ . Personalized health prediction AI models using transfer learning and strategic overfitting on wearable device data. J Med Syst. (2025) 49:45. doi: 10.1007/s10916-025-02180-5, 40199790

[37] Collin CB Gebhardt T Golebiewski M Karaderi T Hillemanns M Khan FM . Computational models for clinical applications in personalized medicine-guidelines and recommendations for data integration and model validation. J Pers Med. (2022) 12:166. doi: 10.3390/jpm12020166, 35207655 PMC8879572

[38] Khalid S Yang C Blacketer C Duarte-Salles T Fernández-Bertolín S Kim C . A standardized analytics pipeline for reliable and rapid development and validation of prediction models using observational health data. Comput Methods Prog Biomed. (2021) 211:106394. doi: 10.1016/j.cmpb.2021.106394, 34560604 PMC8420135

[39] Baião AR Cai Z Poulos RC Robinson PJ Reddel RR Zhong Q . A technical review of multi-omics data integration methods: from classical statistical to deep generative approaches. Brief Bioinform. (2025) 26:bbaf355. doi: 10.1093/bib/bbaf355 40748323 PMC12315550

[40] Adam E Zanoaga MD Rota R Cominetti O. A comprehensive protocol and step-by-step guide for multi-omics integration in biological research. J Vis Exp. (2025):222. doi: 10.3791/66995, 40853860

[41] Lodewyk K Wiebe M Dennett L Larsson J Greenshaw A Hayward J. Wearables research for continuous monitoring of patient outcomes: a scoping review. PLOS Digit Health. (2025) 4:e0000860. doi: 10.1371/journal.pdig.0000860, 40343891 PMC12063813

[42] Dinesh K Snyder CW Xiong M Tarolli CG Sharma S Dorsey ER . A longitudinal wearable sensor study in Huntington's disease. J Huntingtons Dis. (2020) 9:69–81. doi: 10.3233/JHD-190375, 31868675

[43] Farabolini G Baldini N Pagano A Andrenelli E Pepa L Morone G . Continuous movement monitoring at home through wearable devices: a systematic review. Sensors (Basel). (2025) 25:4889. doi: 10.3390/s25164889, 40871752 PMC12389529

[44] Rajkomar A Dean J Kohane I. Machine learning in medicine. N Engl J Med. (2019) 380:1347–58. doi: 10.1056/NEJMra1814259, 30943338

[45] Kawamoto K Houlihan CA Balas EA Lobach DF. Improving clinical practice using clinical decision support systems: a systematic review of trials to identify features critical to success. BMJ. (2005) 330:765. doi: 10.1136/bmj.38398.500764.8F, 15767266 PMC555881

[46] Meng J Wu M Shi F Xie Y Wang H Guo Y. Medical laboratory data-based models: opportunities, obstacles, and solutions. J Transl Med. (2025) 23:823. doi: 10.1186/s12967-025-06802-x, 40707923 PMC12291381

[47] You J Seok HS Kim S Shin H. Advancing laboratory medicine practice with machine learning: swift yet exact. Ann Lab Med. (2025) 45:22–35. doi: 10.3343/alm.2024.0354, 39587856 PMC11609717

[48] Bogaty C Frambach J. The CanMEDS competency framework in laboratory medicine: a phenomenographic study exploring how professional roles are applied outside the clinical environment. Can Med Educ J. (2024) 15:26–36. doi: 10.36834/cmej.77140, 38528898 PMC10961121

[49] Oosterhuis W. Adding clinical utility to the laboratory reports: automation of interpretative comments. Clin Chem Lab Med. (2019) 57:365–70. doi: 10.1515/cclm-2018-0623, 30367781

[50] Plebani M. Interpretative commenting: a tool for improving the laboratory-clinical interface. Clin Chim Acta. (2009) 404:46–51. doi: 10.1016/j.cca.2009.03.012, 19298798

[51] Pillay TS Topcu Dİ Yenice S. Harnessing AI for enhanced evidence-based laboratory medicine (EBLM). Clin Chim Acta. (2025) 569:120181. doi: 10.1016/j.cca.2025.120181, 39909187

[52] Zhao Q Zhang C Zhang W Zhang S Liu Q Guo Y. Applications and challenges of biomarker-based predictive models in proactive health management. Front Public Health. (2025) 13:1633487. doi: 10.3389/fpubh.2025.1633487, 40900695 PMC12399543

[53] Arora A Alderman JE Palmer J Ganapathi S Laws E McCradden MD . The value of standards for health datasets in artificial intelligence-based applications. Nat Med. (2023) 29:2929–38. doi: 10.1038/s41591-023-02608-w, 37884627 PMC10667100

[54] Teng Q Liu Z Song Y Han K Lu Y. A survey on the interpretability of deep learning in medical diagnosis. Multimed Syst. (2022) 28:2335–55. doi: 10.1007/s00530-022-00960-4, 35789785 PMC9243744

[55] Yang J Soltan AAS Clifton DA. Machine learning generalizability across healthcare settings: insights from multi-site COVID-19 screening. NPJ Digit Med. (2022) 5:69. doi: 10.1038/s41746-022-00614-9, 35672368 PMC9174159

[56] Wainstein M Flanagan E Johnson DW Shrapnel S. Systematic review of externally validated machine learning models for predicting acute kidney injury in general hospital patients. Front Nephrol. (2023) 3:1220214. doi: 10.3389/fneph.2023.1220214, 37675372 PMC10479567

[57] Paulovich AG Whiteaker JR Hoofnagle AN Wang P. The interface between biomarker discovery and clinical validation: the tar pit of the protein biomarker pipeline. Proteomics Clin Appl. (2008) 2:1386–402. doi: 10.1002/prca.200780174, 20976028 PMC2957839

[58] Walsh CG McKillop MM Lee P Harris JW Simpson C Novak LL. Risky business: a scoping review for communicating results of predictive models between providers and patients. JAMIA Open. (2021) 4:ooab092. doi: 10.1093/jamiaopen/ooab092, 34805776 PMC8598291

[59] Zhang P Kamel Boulos MN. Privacy-by-design environments for large-scale Health Research and federated learning from data. Int J Environ Res Public Health. (2022) 19:11876. doi: 10.3390/ijerph191911876, 36231175 PMC9565554

[60] Cestonaro C Delicati A Marcante B Caenazzo L Tozzo P. Defining medical liability when artificial intelligence is applied on diagnostic algorithms: a systematic review. Front Med (Lausanne). (2023) 10:1305756. doi: 10.3389/fmed.2023.1305756, 38089864 PMC10711067

[61] Shen J Andersen R Brook R Kominski G Albert PS Wenger N. The effects of payment method on clinical decision-making: physician responses to clinical scenarios. Med Care. (2004) 42:297–302. doi: 10.1097/01.mlr.0000114918.50088.1c, 15076830

[62] Meunier PY Raynaud C Guimaraes E Gueyffier F Letrilliart L. Barriers and facilitators to the use of clinical decision support Systems in Primary Care: a mixed-methods systematic review. Ann Fam Med. (2023) 21:57–69. doi: 10.1370/afm.2908, 36690490 PMC9870646

[63] Watson J Hutyra CA Clancy SM Chandiramani A Bedoya A Ilangovan K . Overcoming barriers to the adoption and implementation of predictive modeling and machine learning in clinical care: what can we learn from US academic medical centers? JAMIA Open. (2020) 3:167–72. doi: 10.1093/jamiaopen/ooz046, 32734155 PMC7382631

[64] Flores E Salinas JM Blasco Á López-Garrigós M Torreblanca R Carbonell R . Clinical decision support systems: a step forward in establishing the clinical laboratory as a decision maker hubA CDS system protocol implementation in the clinical laboratory. Comput Struct Biotechnol J. (2023) 22:27–31. doi: 10.1016/j.csbj.2023.08.006, 37661968 PMC10474568

[65] Li X Huang L Zhang H Liang Z. Enabling telemedicine from the system-level perspective: scoping review. J Med Internet Res. (2025) 27:e65932. doi: 10.2196/65932, 40053725 PMC11923472

[66] Kaboré SS Ngangue P Soubeiga D Barro A Pilabré AH Bationo N . Barriers and facilitators for the sustainability of digital health interventions in low and middle-income countries: a systematic review. Front Digit Health. (2022) 4:1014375. doi: 10.3389/fdgth.2022.1014375, 36518563 PMC9742266

[67] Scott Kruse C Karem P Shifflett K Vegi L Ravi K Brooks M. Evaluating barriers to adopting telemedicine worldwide: a systematic review. J Telemed Telecare. (2018) 24:4–12. doi: 10.1177/1357633X16674087, 29320966 PMC5768250

[68] Yi S Yam ELY Cheruvettolil K Linos E Gupta A Palaniappan L . Perspectives of digital health innovations in low- and middle-income health care systems from south and Southeast Asia. J Med Internet Res. (2024) 26:e57612. doi: 10.2196/57612, 39586089 PMC11629033

[69] Collins GS Moons KGM Dhiman P Riley RD Beam AL Van Calster B . TRIPOD+AI statement: updated guidance for reporting clinical prediction models that use regression or machine learning methods. BMJ. (2024) 385:e078378. doi: 10.1136/bmj-2023-078378, 38626948 PMC11019967

[70] Liu X Cruz Rivera S Moher D Calvert MJ Denniston AK SPIRIT-AI and CONSORT-AI Working Group. Reporting guidelines for clinical trial reports for interventions involving artificial intelligence: the CONSORT-AI extension. Lancet Digit Health. (2020) 2:e537–48. doi: 10.1016/S2589-7500(20)30218-1, 33328048 PMC8183333

[71] Yang Y Cui YU Wang YT Xue P Zhai XM Qiao YL. Interpretation of the WHO'S "ethics and governance of artificial intelligence for health: guidance on large multi-modal models" and its implications for China. Zhonghua Yu Fang Yi Xue Za Zhi. (2025) 59:6, 960–969. Chinese. doi: 10.3760/cma.j.cn112150-20240709-00548 40518431

[72] Abràmoff MD Roehrenbeck C Trujillo S Goldstein J Graves AS Repka MX . A reimbursement framework for artificial intelligence in healthcare. NPJ Digit Med. (2022) 5:72. doi: 10.1038/s41746-022-00621-w, 35681002 PMC9184542

[73] Misra R Keane PA Hogg HDJ. How should we train clinicians for artificial intelligence in healthcare? Future Healthc J. (2024) 11:100162. doi: 10.1016/j.fhj.2024.100162, 39371537 PMC11452832

[74] Winslow RL Trayanova N Geman D Miller MI. Computational medicine: translating models to clinical care. Sci Transl Med. (2012) 4:158rv11. doi: 10.1126/scitranslmed.3003528, 23115356 PMC3618897

[75] Yadalam AK Liu C Hui Q Razavi AC Sperling LS Quyyumi AA . Large-scale proteomics-based risk score for the prediction of incident cardio-kidney-metabolic disease risk. Circ Genom Precis Med. (2025) 18:e005125. doi: 10.1161/CIRCGEN.124.005125, 40931818 PMC12554283

[76] Carrasco-Zanini J Pietzner M Davitte J Surendran P Croteau-Chonka DC Robins C . Proteomic signatures improve risk prediction for common and rare diseases. Nat Med. (2024) 30:2489–98. doi: 10.1038/s41591-024-03142-z, 39039249 PMC11405273

[77] Liu Y Ritchie SC Teo SM Ruuskanen MO Kambur O Zhu Q . Integration of polygenic and gut metagenomic risk prediction for common diseases. Nat Aging. (2024) 4:584–94. doi: 10.1038/s43587-024-00590-7, 38528230 PMC11031402

[78] Gao P Shen X Zhang X Jiang C Zhang S Zhou X . Precision environmental health monitoring by longitudinal exposome and multi-omics profiling. Genome Res. (2022) 32:1199–214. doi: 10.1101/gr.276521.121, 35667843 PMC9248886

[79] Denny JC. Enabling a healthier future for all through precision medicine. Trans Am Clin Climatol Assoc. (2025) 135:60–73. 40771618 PMC12323482

[80] L'Hommedieu M L'Hommedieu J Begay C Schenone A Dimitropoulou L Margolin G . Lessons learned: recommendations for implementing a longitudinal study using wearable and environmental sensors in a health care organization. JMIR Mhealth Uhealth. (2019) 7:e13305. doi: 10.2196/13305, 31821155 PMC6930504

[81] Roberts MC Holt KE Del Fiol G Baccarelli AA Allen CG. Precision public health in the era of genomics and big data. Nat Med. (2024) 30:1865–73. doi: 10.1038/s41591-024-03098-0, 38992127 PMC12017803

[82] Gambhir SS Ge TJ Vermesh O Spitler R Gold GE. Continuous health monitoring: an opportunity for precision health. Sci Transl Med. (2021) 13:eabe5383. doi: 10.1126/scitranslmed.abe5383, 34108250

[83] Enticott J Johnson A Teede H. Learning health systems using data to drive healthcare improvement and impact: a systematic review. BMC Health Serv Res. (2021) 21:200. doi: 10.1186/s12913-021-06215-8, 33663508 PMC7932903

[84] Buckeridge DL. Precision, equity, and public health and epidemiology informatics-a scoping review. Yearb Med Inform. (2020) 29:226–30. doi: 10.1055/s-0040-1701989, 32823320 PMC7442517

